# Improvements to the Nutri-Score to address challenges identified in a Nordic setting

**DOI:** 10.29219/fnr.v69.10914

**Published:** 2025-01-29

**Authors:** Anna Amberntsson, Mari Mohn Paulsen, Marta Angela Bianchi, Bryndís Eva Birgisdóttir, Anja Pia Biltoft-Jensen, Dina Moxness Konglevoll, Anne Lise Brantsæter, Kaja Lund-Iversen, Lene Frost Andersen, Marianne Hope Abel

**Affiliations:** 1 Department of Food Safety, Norwegian Institute of Public Health, Oslo, Norway; 2 Centre for Sustainable Diets, Norwegian Institute of Public Health, Oslo, Norway; 3 Department of Agriculture and Food, RISE Research Institutes of Sweden, Gothenburg, Sweden; 4 Unit for Nutrition Research, Faculty of Food Science and Nutrition, School of Health Sciences, University of Iceland, Reykjavík, Iceland; 5 National Food Institute, Technical University of Denmark, Copenhagen, Denmark; 6 Department of Nutrition, Institute of Basic Medical Sciences, University of Oslo, Oslo, Norway; 7 Research Administrative Support, Norwegian Institute of Public Health, Oslo, Norway; 8 Department of Physical Health and Ageing, Norwegian Institute of Public Health, Oslo, Norway; 9 Centre for Evaluation of Public Health Measures, Norwegian Institute of Public Health, Oslo, Norway

**Keywords:** nutrient profiling, front-of-pack nutrition label, nutritional quality, nordic nutrition recommendations, carbohydrate quality, fat quality, public health policy, food classification

## Abstract

**Background:**

Front-of-pack nutrition labelling is an important policy tool for public health. The Nutri-Score classifies foods according to nutritional quality from A (high quality) to E (low quality). We have previously identified inconsistencies between Nutri-Score and the Norwegian food-based dietary guidelines. The objective was to propose revisions to the Nutri-Score 2023 algorithms and determine if the revised algorithms better align with the Nordic Nutrition Recommendations 2023 (NNR2023) and the Keyhole label.

**Methods:**

Items in the Norwegian pre-packed foods databases Tradesolution (*n* = 26,033) and Unil (*n* = 577) were classified using the Nutri-Score 2023 algorithms. To address carbohydrate quality, a penalty for low-fibre content was introduced, and the sugar scale compressed. The protein cap was removed for fish products to reward their nutritional quality. To improve the scoring of high-fat foods, the scale for saturated fat was extended, fat content determined the inclusion in the algorithm for fats, rather than food categories, and favourable fat quality in oils was rewarded through a fat quality component. Data from the databases guided the identification of specific thresholds. The distribution of Nutri-Score was calculated before and after applying the revisions.

**Results:**

In total, 5.5% of all products received a less favourable Nutri-Score with the revised carbohydrate quality components. Most refined pastas and flour shifted shifted from A to B or C, whilst whole grain pasta largely remained A. Sugar-rich breakfast cereals shifted from B to C or D. For fish, 11% (1% of all products) were moved from D or E to C or D. The variation in scores for cheese and creams increased. Around 5% of all products were affected by the revisions related to fat quality.

**Conclusions:**

The proposed revisions make the Nutri-Score more coherent with the NNR2023 and the Keyhole label. The proposed revisions also hold relevance for other European countries and should therefore be considered in the next revision of the Nutri-Score.

## Popular scientific summary

- The Nutri-Score guides consumers towards healthier food choices but fails within specific categories.
- The Nutri-Score is considered as the harmonised front-of-pack nutrition label in the European Union.
- This study proposes seven revisions to improve the Nutri-Score’s classification of carbohydrate-rich foods, fish and fat-rich foods.
- To improve the overall performance of the Nutri-Score and enhance the relevance of the nutrition label in the Nordic countries, the proposed revisions should be implemented in the next update of the algorithm.

Nutrient profiling models have been recognised as an important policy instrument for public health (1). It is defined as ‘the science of classifying or ranking foods according to their nutritional composition for reasons related to preventing disease and promoting health’ (2). One common use of nutrient profiling models has been as front-of-pack nutrition labelling, aiming to guide consumers towards making healthier dietary choices (3, 4), whilst concurrently encouraging manufacturers to nutritionally improve their products. The European Union’s (EU) current regulation on food information to consumers stipulates that front-of-pack nutritional labels are optional, allowing food manufacturers the discretion to decide whether to include them on their products (5). However, as part of the Farm to Fork strategy adopted by the European Commission in 2020, the EU plans to revise this regulation in the coming years to introduce a single, standardised front-of-pack nutrition label that will be mandatory across all member states and the European Economic Area (6).

## The NewTools project

NewTools is a research project led by the Norwegian Institute of Public Health, aiming to develop two scoring systems for foods; one that indicates nutritional quality, and one that indicates environmental and social sustainability (7). The overall goal is to contribute to a transformation of the Norwegian food system to become more sustainable through research and collaboration with Norwegian food system actors, including governmental bodies, food industry and civil society. Previous literature has emphasised the importance of adopting or adapting established nutrient profiling models to ensure standardised and evidence-based practices in the field and minimise the risks of confusion, inconsistencies between models and potential loss of credibility amongst regulators and consumers (2, 8). In the work with the scoring system for nutritional quality of foods, it was decided to use the existing nutrient profiling model Nutri-Score as a starting point (9, 10). Nutri-Score had already been investigated thoroughly and been shown to classify foods by their nutritional quality coherently with nutritional recommendations (11–14). Moreover, amongst nutrient profiling systems currently in use in the EU, Nutri-Score was the one best fitting to the pre-defined criteria and aspirations for the scoring system specified in the NewTools project steering document (see Methods section).

## The Nutri-Score

The Nutri-Score is a nutrient profiling model and a front-of-pack nutrition label used to demonstrate the overall nutritional quality of food products ranging from A (highest nutritional quality) to E (lowest nutritional quality) (15, 16). It has been adopted by France, Belgium, Germany, Luxembourg, the Netherlands, Spain and Switzerland since its launch in 2017. The Nutri-Score has been developed by the French public health agency Santé Publique France in collaboration with various scientific and governmental organisations. The model was based on a nutrient profiling system developed by researchers at the University of Oxford for the British Food Standards Agency (17, 18). In 2022–2023, the scientific committee of the Nutri-Score updated the algorithms (henceforth referred to as the Nutri-Score 2023 algorithms) to better align with food-based dietary guidelines of the countries that have adopted the label (10, 19). Nutri-Score consists of three different algorithms: the main algorithm for general foods, the algorithm for fats, oils, nuts and seeds, and the algorithm for beverages (Additional file 1).

## The Nordic Nutrition Recommendations and the Keyhole label

The Nordic Nutrition Recommendations (NNR) represent the most up-to-date and comprehensive compilation of research on food and health and is the scientific basis for national dietary guidelines in the Nordic and Baltic countries (20). NNR is regularly updated, and the most recent version was released in 2023 (NNR2023). The Keyhole label is a nutrition label adopted by health authorities in Sweden, Norway, Denmark, Iceland, Lithuania and North Macedonia (21, 22) and notified to the EU as a nutrition claim. Eligibility criteria for displaying the Keyhole label on food items are based on the NNR. The Keyhole label is an endorsement label and signifies that a food item is a healthier choice (contains less salt, sugar, saturated fat and higher amounts of fibre and whole grains) when compared to other items in the same category.

## Identified challenges of the Nutri-Score

As part of the NewTools project, we have scientifically evaluated the Nutri-Score 2023 algorithms in a Norwegian setting (23). In the evaluation, the alignment between Nutri-Score and Norwegian food-based dietary guidelines (based on the NNR) was assessed, as well as the ability to discriminate foods by nutritional quality within food categories. In addition, input was gathered from food system actors represented by partners and followers of the NewTools project on how the Nutri-Score performed in classifying foods in the Norwegian market (24). Although the scientific evaluation revealed an overall acceptable discriminatory performance within most food categories, both the scientific evaluations (23) and the input from the food system actors (24) identified some important inconsistencies with the Nutri-Score and the NNR2023. These inconsistencies were assessed by the research team, and the most important challenges were identified (Table 1).

**Table 1:** Inconsistencies and challenges between the Nutri-Score 2023 algorithms and the Nordic Nutrition Recommendations 2023

Identified challenges	Detailed description
Poor discrimination between whole grain and refined grain products of rice, pasta and flour in the main algorithm for general foods (23–25).	Both whole grain and refined grain products of rice, pasta and flour generally obtained the most favourable Nutri-Score A. This is not in line with the NNR2023 and the Keyhole label, stating that whole grain cereals should preferably be selected (20).
Some breakfast cereals get Nutri-Score B despite a high content of added sugar. Maximum unfavourable points for total sugar are allocated at a content >51 g/100 g.	In the NNR2023, the intake of added sugar is recommended to be limited (16). The Keyhole label only allows for 13 g/100 g of total sugar in breakfast cereals and muesli. In the Nutri-Score main algorithm, a total sugar content of 13 g/100 g is penalised with only 3 (out of 15) unfavourable points for sugar.
Fresh fruits and vegetables without nutritional declaration are not assigned with a Nutri-Score (16).	Only products with a mandatory nutritional declaration are covered by the Nutri-Score. It is problematic that Nutri-Score does not guide consumers towards the healthiest foods in the market.
Nutri-Score does not specifically reward the nutritional quality of fish.	The NNR2023 recommend an intake of 300–450 g fish per week (20). The Keyhole label allows for up to 3 g salt/100 g in smoked and canned fish. Several Keyhole-labelled fish products obtain a poor Nutri-Score.
Nutri-Score does not differentiate between full-fat cheeses and the leaner alternatives (19).	The main algorithm does not capture variation in saturated fat content >10 g/100 g. Most cheese products cluster in Nutri-Score D. The NNR2023 recommend that dairy products containing high levels of saturated fat should be limited.
The algorithm for fats, oils, nuts and seeds does not differentiate between full-fat creams and the leaner alternatives (19). The main algorithm for general foods does not consider the fat quality in high-fat foods.	Creams are included in the Nutri-Score algorithm for fats, oils, nuts and seeds, which are adapted to fatty foods and perform poorly in discriminating products with a low- to medium-fat content (like creams). Most creams cluster in Nutri-Score D. Whilst the algorithm for fats, oils, nuts and seeds performs well for products with a high content of fat, products with a high-fat content that are not included in this algorithm, like, for example, mayonnaise and dressings, obtain a poor score under the main algorithm regardless of fat quality (23).
Oils are not scored equally based on fat quality.	In the algorithm for fats, oils, nuts and seeds, only oils from ingredients qualifying in the list of fruits, vegetables and legumes (FVL) receive favourable points for this component. This action was implemented to reward some specific plant-based oils with favourable fat quality. However, to concur with the NNR2023, all plant-based oils with favourable fat quality should be encouraged (16).

## Research gap and aim

Previous studies have shown an overall acceptable agreement between the Nutri-Score 2023 algorithms and the Norwegian food-based dietary guidelines and the Keyhole label (23, 25). Still, there are some remaining inconsistencies as described in Table 1. These inconsistencies should be addressed for the Nutri-Score to become relevant in a Nordic setting. *As part of the NewTools-project, focusing on inconsistencies related to carbohydrate quality, fish products and scoring of saturated fats and high-fat foods, the objective of this study was to propose revisions to the Nutri-Score 2023 main algorithm for general foods and the algorithm for fats, oils, nuts and seeds and to determine if the revised algorithms better align with the NNR2023 and the Keyhole label.*

## Methods

### Framework for the proposed revisions to the Nutri-Score 2023 algorithms

The overall ambition of the score revised within the NewTools project is to classify foods based on the nutritional quality. A framework was specified, consisting of a set of criteria and aspirations to guide the revision process. These criteria stated that the score:

- is not a tool measuring the total healthiness of foods or diets.
- should be suitable as a basis for front-of-pack nutrition labelling, aiming at guiding consumers towards healthier food choices.
- can be useful as a tool in different parts of the food system, such as guiding product development/reformulation and regulations for marketing of food to children.
- is developed by researchers.
- must be evidence based.
- should coincide with the definition of a nutrient profile model (26).
- combines relevant components into one single-scale summary score.
- strives to only include components that are easily accessible for the producers.
- may include components not mandatory to declare on prepacked foods according to EU labelling rules (27).
- is not more complex than it needs to be.
- should align well with the NNR2023 (20).
- should provide variation in scores within each food category to be able to guide towards healthier choices.

Whilst NNR2023 was the guiding reference document for the revision process of the Nutri-Score, data from the food databases (as described below) guided the identification of specific cut-offs and thresholds to ensure that the variation in the components of the score was sufficiently captured. In addition to the Norwegian research team, researchers from Sweden, Denmark and Iceland with expertise within nutrient profiling were invited as co-authors to contribute with a broader Nordic perspective on the proposed revisions.

### Food databases

We used two food databases in the work with the proposed revisions of Nutri-Score. Tradesolution is a database owned by three retail group companies and Grocery Suppliers of Norway (DLF Norway), a trade association for food manufacturers and suppliers, comprising 40,915 prepacked foods and beverages sold in Norway. Unil is a private label food supply company owned by the Norwegian retail group NorgesGruppen, with a database comprising 770 pre-packed food items from the supplier Unil only. The sample of foods in this study included all items in Tradesolution (version June 2023) and Unil (version November 2022) that were eligible for calculating the Nutri-Score 2023 algorithms (16). After the exclusion of non-foods, beverages, spices and herbs, products with missing information on the components of the Nutri-Score, implausible values and duplicates, the database contained 26,610 foods (26,033 from Tradesolution and 577 from Unil), of which 25,833 qualified for the main algorithm and 777 qualified for the algorithm for fats, oils, nuts and seeds. Beverages were excluded from the database, as no inconsistencies in the scoring of beverages between the Nutri-Score 2023 beverage algorithm and the NNR2023 had been identified (Table 1).

We used the food composition database KBS, version 7.4, AE-22 at the Department of Nutrition, University of Oslo, Norway, for information regarding unpacked food items. The database mainly contains generic foods consumed in Norway. Of the 4,199 foods in the database, 236 were FLV on which Nutri-Score was calculated.

### Nutri-Score calculation

All items in the food databases were classified using the Nutri-Score 2023 main algorithm for general foods or the algorithm for fats, oils, nuts and seeds (10) (Additional file 1). This study does not include any suggested revisions regarding the algorithm for beverages.

Products with missing information on fibre (*N* = 15,863) received 0 points for fibre, with the assumption that foods that contain any significant amount of fibre likely have the information declared on the product. Information regarding content of FVL (%) was not available in the databases. All food items categorised as fruit, vegetables or legumes (*N* = 1,899) were assumed to contain >80% FVL and thereby received the maximum score of 5 points. All other products received 0 points for FVL. Oils from ingredients qualifying for the FVL component (16) were manually assigned points for FVL.

### Proposed revisions

#### Carbohydrate quality – Discrimination between whole grain and refined grain in high-carbohydrate foods

The main goal of this revision was that the main algorithm should encourage whole grain products, especially for rice, flour and pasta, at the cost of refined grain products. However, the revision should not affect fresh fruits, vegetables and potatoes, which should continue to obtain Nutri-Score A or B.

The proposed revision was to penalise a low content of fibre. With limited unfavourable points, rice, pasta and flour will obtain Nutri-Score A regardless of fibre content. Thus, to achieve discrimination between whole grain and refined grains, unfavourable points need to be distributed. However, to target only the products we aimed to separate, we suggest that this penalisation only applies to foods with a high-carbohydrate content. What is considered a high-carbohydrate content was identified from the databases.

#### Carbohydrate quality – Penalise products high in added sugar

The main goal of this revision was that the main algorithm should discourage consumption of products high in added sugar. The revision aimed to target products with a high content of added sugar, whilst products with sugars naturally present (such as fresh fruits) should not be affected by the revision. Breakfast cereals were identified as target foods for the revision.

The proposed solution was to modify the sugar scale in the main algorithm. The algorithm does not separate naturally present sugar from added sugars but uses total sugar as stated on the nutrition declaration. Sugars naturally present in fruit and dairy products were identified using the databases, and the sugar scale was modified above this amount to target added sugar and not the naturally present sugar.

#### Nutri-Score for unprocessed fruits and vegetables

The goal of this revision was that the Nutri-Score should be applicable to all unprocessed fruits and vegetables, to encourage intake of these foods.

We propose to use the same definition as applied to the nutrient profiling system and front-of-pack nutrition label The Health Star Rating system: *All whole fresh fruit (except coconut) and vegetables, fungi and legumes (except peanuts) as sold with no processing, plus these same products that have only been peeled, cut and/or surface treated and/or blanched and/or frozen (not dried), or canned without the addition of fat, sugars/sweeteners or salt* (28). Coconut is according to the Nutri-Score documentation considered a nut (16) and is therefore not covered by this revision. We propose that all foods covered by the definition should receive Nutri-Score A and are allocated with a nutritional score of −5, which was the median score amongst all unprocessed fruits and vegetables in the KBS database.

#### Rewarding the nutrient quality of fish products in the main algorithm

The main goal of this revision was to encourage increased consumption of fish. The revision aimed to target products with a high-content of fish, whilst products with a low-fish content should not benefit from the revision. Furthermore, Keyhole-labelled fish products should mainly obtain Nutri-Score C or better.

The proposed solution was to allow all products containing at least 50% fish to receive favourable points for protein even when the number of unfavourable points exceeds 11, like the exemption to the rule made for cheese (10, 16). This was a targeted revision that would not affect any other food product, and it is based on information easily accessible for the food producers.

#### Scoring of high-fat foods – Improve discrimination of products high in saturated fat

The goal of this revision was to better differentiate between products with a high amount of saturated fat in the main algorithm whilst not affecting products with ≤10 g saturated fat/100 g. Cheeses fulfilling the criteria for the Keyhole label (≤17% fat and ≤1.6% salt) should ideally obtain a score C or better. Regular fat cheeses should remain at score D, and very high-fat cheeses should mainly obtain a Nutri-Score E.

The proposed solution was to extend the range for the penalisation of saturated fat to a higher content and extend the number of unfavourable points in the algorithm.

#### Scoring of high-fat foods – Replace the algorithm for fats, oils, nuts and seeds with an algorithm for all high-fat foods

One goal of this revision was to increase the variation in scores for creams, sour creams and their plant-based analogues. A second goal was to achieve a better classification of *all* products with a high-fat content. Finally, the revision should not affect the scoring of most products already included in the oils, fats, nuts and seeds algorithm, except for creams.

There are products with a very high-fat and energy content that are not included in the definition for fats, oils, nuts and seeds. Examples of such products are mayonnaise and dressings. Although these products may have a favourable fat quality, they get a poor Nutri-Score in the main algorithm due to the high energy content. In contrast, products with lower fat content, like creams, should not be included in the algorithm for fats. We propose that the inclusion of products in the algorithm for high-fat foods should be based on the total content of fat and not by type of food.

#### Scoring of high-fat foods – Rewarding oils high in unsaturated fat in the algorithm for high-fat foods

One goal was to improve the classification of all plant-based oils rich in unsaturated fats. The second goal was that products high in saturated fat should not benefit from the revision.

In the algorithm for fats, oils, nuts and seeds, the favourable FVL component is only included in oils made from FVL. In practice, the FVL component favours only some plant-based oils with beneficial nutrient quality, such as olive oil. In the NNR2023, there is no specific recommendations regarding the origin of the fat, only regarding fat quality (20). Thus, oils with similar fat quality should be scored equally. We propose removal of the FVL component in the algorithm for high-fat foods, in favour of a new fat quality component, which gives favourable points for a low content of saturated fats (and thereby a high content of unsaturated fats). To target only the products we aimed to separate, we suggest that this penalisation only applies to oils.

### Ethics

Ethical approval was not required as no human or animal subjects were involved in this study.

### Statistical analysis

Histograms were used to display the distribution of nutrients in products, which were used to identify cut-offs for total carbohydrates and total fat. Histograms were also used to display the distribution of Nutri-Score within food categories before and after applying the proposed revisions. The distribution (in percent) and mean Nutri-Score, calculated as A–E corresponding to 1–5, were also presented before and after applying the proposed revisions. Energy percentage from protein ((Protein (g/100 g) * 17)/total energy content (kJ/100 g)) was used as a proxy for the percentage of fish in products classified as fish in the databases. Statistical analyses were performed using Stata (StataCorp Stata Statistical Software: Release 17, College Station, TX, USA).

## Results

### Carbohydrate quality

#### Discrimination between whole grain and refined grain in high-carbohydrate foods in the main algorithm

**Fig. 1:**
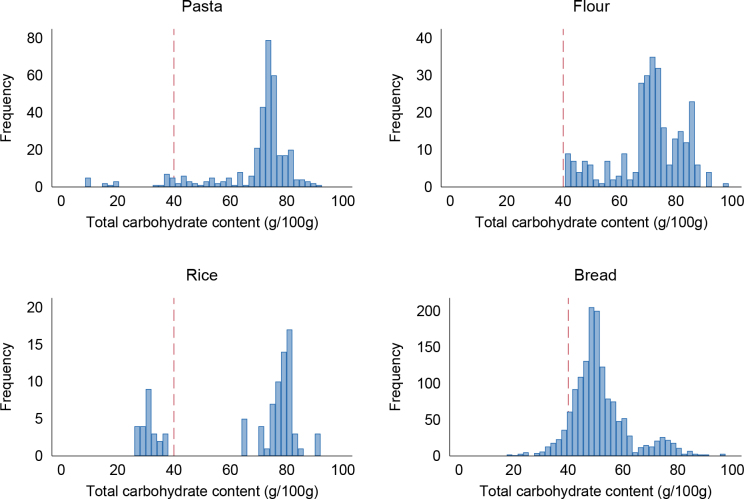
Total carbohydrate content (including fibre) in pasta (*N* = 605), flour (*N* = 425), rice (*N* = 170) and bread (*N* = 1,200). Dotted red line indicates 40 g total carbohydrate/100 g. Frequency is the number of products. The databases are Tradesolution and Unil.

First, we identified a suitable cut-off to define high-carbohydrate foods. When inspecting the total carbohydrate content (including starch, sugar and fibre), most of the rice, pasta, flour and bread had a total carbohydrate content ≥40 g/100 g (Fig. 1). This was selected as the cut-off to define high-carbohydrate foods to be penalised for a low-fibre content.

Second, we suggest that the foods with ≥40 g carbohydrates/100 g will not be scored with the original Nutri-Score fibre scale but will have an adapted scoring for fibre. When investigating the fibre content in products with >40 g total carbohydrates/100 g in the databases, most refined grain products had a fibre content <3.5 g/100 g (75^th^ percentile). Therefore, products with a fibre content of <3.5 g/100 g should receive unfavourable points for fibre to be moved to a more neutral Nutri-Score. However, to align with the original fibre scale, 4.1 g fibre/100 g was selected as a suitable cut-off for introduction of the unfavourable points for fibre, since this is the current cut-off point between 1 and 2 points. The proposed new fibre scale for carbohydrate-rich foods penalises a low-fibre content with 2–3 points to be able to move food items from A to B or C. Unfavourable points were allocated for products with ≤4.1 g fibre/100 g, whilst products with >4.1 g fibre/100 g get points according to the original fibre scale (Table 2).

**Table 2:** Proposed revision of the favourable fibre component in the main algorithm, for foods with a total carbohydrate content ≥40 g/100 g only

Points	Fibre (g/100 g)	
	Current algorithm	Proposed revision
-3	-	≤2.0
-2	-	>2
0	≤3.0	-
1	>3.0	-
2	>4.1	>4.1
3	>5.2	>5.2
4	>6.3	>6.3
5	>7.4	>7.4

The databases are Tradesolution and Unil.

In the databases, 4,444 products (16%) of the general foods had a total carbohydrate content of ≥40 g/100 g (Table 3). However, only 669 (15%) of those products obtained a new and more unfavourable Nutri-Score. The products that mainly were affected by this revision were cereals/grains, rice and pasta, dried and canned fruit and berries, some refined bread products, and some sandwich toppings (jam, etc.). Amongst all products with ≥40 g carbohydrates/100 g, there were no products with missing information on fibre.

**Table 3:** Distribution (%) of the target food groups with ≥40 g total carbohydrates (*N* = 4,282) in the current (left) and proposed revision (right) for fibre in the main algorithm

Food group	Nutri-Score (%) Current algorithm							Nutri-Score (%) Proposed revision					
	N	NS	A	B	C	D	E	NS	A	B	C	D	E
Refined pasta	284	1.9	50	29	11	6	4	2.6	8	41	40	7	4
Whole grain pasta^a^	39	1.1	92	8	0	0	0	1.3	85	5	10	0	0
Refined flour	189	2.2	51	10	18	15	6	2.6	27	22	29	12	10
Whole grain flour^a^	83	1.1	95	2	3	0	0	1.1	93	2	5	0	0
Refined rice	56	2.3	0	80	4	16	0	3.2	0	0	84	16	0
Whole grain rice^a^	5	1.6	40	60	0	0	0	2.6	20	0	80	0	0
Refined grain bread	813	2.7	11	19	59	10	1	2.8	10	16	62	11	1
Other breads	107	3.1	7	12	51	23	7	3.3	2	12	54	19	13
Whole grain bread^a^	201	1.5	63	26	7	4	0	1.5	63	26	7	4	0
Breakfast cereals	163	2.4	33	13	35	15	4	2.5	33	13	34	15	5
Quinoa, bulgur, couscous	28	1.2	89	7	0	4	0	1.3	79	14	3	4	0
Sweet snacks and desserts	1,245	4.5	3	1	6	24	66	4.6	2	0	7	18	73
Biscuits, cakes, pastries, muslibars	379	3.9	1	1	25	48	25	4.0	0	0	24	46	30
Sweet sandwich toppings (jam, etc.)	160	4.2	1	2	2	68	27	4.5	1	0	4	39	56
Fruit and berries^b^	125	3.4	0	2	60	35	3	4.0	0	0	14	67	19
Legumes^b^	75	1.3	91	0	1	4	4	1.4	85	5	2	0	8
Sauce and dressing	44	4.8	0	0	2	16	82	4.9	0	0	2	4	94
Salty snacks	273	3.9	1	1	15	67	16	4.1	1	1	13	60	25
Ready meals	42	3.5	6	12	25	39	18	3.6	4	8	29	39	20

NS, Mean Nutri-Score (A-E = 1–5). Lower value indicates higher nutritional quality. The databases are Tradesolution and Unil.

a Whole grain defined as the first ingredient being whole grain or being eligible for the Keyhole label.

b Dried or canned.

When adapting the suggested revision of the main algorithm, most whole grain pastas remained at Nutri-Score A, whilst most refined pasta were moved from A to B or C (Table 3). Some whole grain pastas were moved to Nutri-Score C, which were pasta made of whole grain rice (gluten free pasta) and with a low-fibre content (2.5–3.2 g/100 g). Of the refined pastas, 8% still got Nutri-Score A due to many favourable points from a high-protein content. Whole grain flour was mainly unaffected by the revision and remained at score A, whilst refined flour (such as wheat flour) was moved from Nutri-Score A to B or C. Regarding rice, four of the five whole grain rice products in the database moved from A or B to C, due to their relatively low-fibre content (1.9–4.0 g/100 g), whilst one whole grain rice maintained its class (A) and had a higher fibre content at 9 g/100 g. Refined rice was previously mainly categorised as Nutri-Score B, but with the revised fibre algorithm, these products were moved to Nutri-Score C. Whole grain bread was unaffected, whilst around 5% of the refined bread products were moved one class lower.

Fresh fruits, vegetables and potatoes have a total carbohydrate content <40 g/100 g and were therefore not affected by the revision. Products not targeted by the revision (<40 g carbohydrates/100 g) but categorised in the same food categories as those with ≥40 g carbohydrates/100 g and their Nutri-Score are presented in Supplementary Table 1 in Additional file 2.

#### Penalising products high in added sugar

First, we inspected the content of total sugar in fruits and dairy products with no added sugar according to the ingredients list. Most of the fruits and dairy products had a natural sugar content ≤12 g/100 g. This was selected as the cut-off below which no changes in the sugar scale would be made. Second, we suggest that the sugar scale should be modified above this cut-off, to penalise added sugar content more heavily (Table 4).

**Table 4:** Proposed revision of the unfavourable sugar component in the main algorithm

Points	Sugar (g/100 g)	
	Current algorithm	Proposed revision
0	≤3.4	≤3.4
1	>3.4	>3.4
2	>6.8	>6.8
3	>10	>10
4	>14	>12
5	>17	>14
6	>20	>16
7	>24	>18
8	>27	>20
9	>31	>22
10	>34	>24
11	>37	>26
12	>41	>28
13	>44	>30
14	>48	>32
15	>51	>34

In the databases, 6,509 products (24%) of the general foods had a total sugar content >12 g/100 g. However, only 890 (3%) of all products in the database obtained a new and more unfavourable Nutri-Score (Table 5). The products that mainly were affected by this revision were sweet snacks and desserts, sweet sandwich toppings (jam, etc.), biscuits and cakes, and breakfast cereals.

**Table 5:** Distribution (%) of Nutri-Score of the target food groups with >12 g sugar/100 g (*N* = 6,509) in the current (left) and proposed revision (right) in the main algorithm

Food group	N	Nutri-Score (%) Current algorithm						Nutri-Score (%) Proposed revision					
		NS	A	B	C	D	E	NS	A	B	C	D	E
Sweet snacks and desserts	4,245	4.7	0	0	6	22	72	4.8	0	0	4	16	80
Sweet sandwich toppings	575	3.8	0	0	24	72	3	4.0	0	0	4	92	4
Biscuits and cakes	417	4.3	0	0	14	47	39	4.4	0	0	7	46	47
Sauces and dressings	389	4.6	0	0	6	32	62	4.7	0	0	4	21	75
Breakfast cereals	96	3.3	5	12	46	28	9	3.6	2	3	38	43	14
Fruit and berries	235	3.0	18	7	37	35	3	3.1	17	3	39	38	4
Vegetables and legumes^a^	91	2.8	22	21	21	25	11	3.1	20	10	29	27	14
Fish products ^a^	161	4.8	0	0	1	19	80	4.9	0	0	0	10	90
Breads	44	3.9	2	0	19	63	16	4.2	0	2	5	63	30
Cheese	72	4.6	0	0	11	21	68	4.7	0	0	6	19	75
Yoghurt	41	3.3	0	3	75	11	11	3.4	0	0	76	13	11
Salty snacks	93	4.6	0	0	8	25	67	4.8	0	0	2	17	81
Meat products	20	4.8	0	0	0	16	84	4.9	0	0	0	5	95
Ready meals	25	4.1	0	0	20	48	32	4.3	0	0	16	40	44

NS, Mean Nutri-Score (A-E = 1–5). Lower value indicates higher nutritional quality. The databases are Tradesolution and Unil.

a Pickled products.

When adapting the suggested revision of the main algorithm, the food items from the category fruits and berries moving to a poorer Nutri-Score were canned fruit in juice or syrup (*N* = 21) and dried fruit (*N* = 9), of which only three had no added sugar and were moved from C to D. Most of the dried fruits with >12 g sugar/100 g in the database (*N* = 130) were not affected by the revision. Vegetables moved to a poorer score were mainly pickled or fermented vegetables with added sugar (*N* = 16). Breakfast cereals moved to a poorer Nutri-Score were mainly crunchy muesli and other sugar-rich cereals (*N* = 30). However, some muesli (*N* = 6) with a high content of dried fruits and berries, with no added sugar, were also moved to a poorer score (from B to C or from C to D).

### Nutri-Score for unprocessed fruits and vegetables

The proposed revision will automatically assign all unprocessed and minimally processed fruits and vegetables according to the proposed definition with a nutritional score of −5 and correspondingly Nutri-Score A.

### Rewarding the nutrient quality of fish products in the main algorithm

**Fig. 2:**
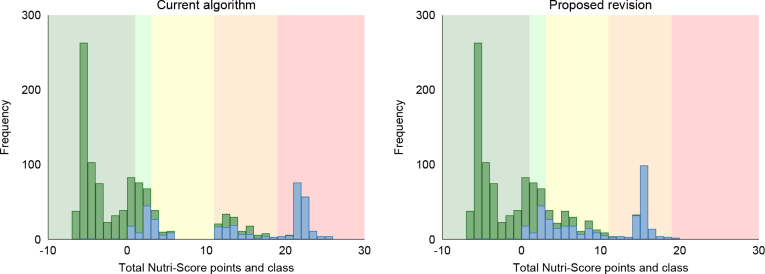
Total Nutri-Score points and class in fish in the main algorithm (left) and after receiving the favourable protein points for fish products with ≥50% fish (right). Frequency is the number of products. The databases are Tradesolution and Unil (*N* = 1,209). Green bars represent fish products fulfilling the criteria for the Keyhole label, and blue bars represent fish products not fulfilling the Keyhole criteria (21).

We propose to remove the protein cap for all fish products containing at least 50% fish, which allows for these products to receive the favourable points for protein even when the number of unfavourable points exceeds 11. Of the 2,647 fish products in the database, 1,209 (45%) had ≥50% fish (here defined as energy from protein ≥50%). Among all fish products, 298 (11%) got a better Nutri-Score after the revision when receiving the favourable protein points, of which 162 were moved from E to D, and 136 were moved from D to C (Fig. 2, Table 6). Only 12 products fulfilling the Keyhole label criteria (21) still got a Nutri-Score D, but no Keyhole labelled product scored E.

**Table 6:** Distribution (%) of the target food groups with ≥50% fish (*N* = 1,209) in the current (left) and proposed revision (right) in the main algorithm

Food group	N	Nutri-Score (%) Current algorithm						Nutri-Score (%) Proposed revision					
		NS	A	B	C	D	E	NS	A	B	C	D	E
Fresh or minimally processed fish	840	1.5	74	13	4	8	1	1.4	74	13	11	1	0
Smoked/cured fish	112	3.3	15	15	9	46	15	2.7	15	15	54	15	1
Canned/pickled fish	89	4.2	12	7	3	2	76	3.4	12	7	6	75	1
Dried fish	93	4.0	9	10	11	12	58	3.3	9	10	23	58	0
Other fish products (fish cakes/pudding, etc.)	52	2.5	35	21	14	15	15	2.2	35	21	29	15	0

NS, Mean Nutri-Score (A-E = 1–5). Lower value indicates higher nutritional quality. The databases are Tradesolution and Unil.

### Scoring of high-fat foods

The suggested revision for fat consisted of three parts, described below.

#### Improve discrimination in products high in saturated fat

We suggest that the range for saturated fat content is penalised up to 20 g/100 g instead of 10 g/100 g, and that the maximum number of unfavourable points is increased from 10 to 15 (Table 7).

**Table 7:** Proposed revision of the unfavourable saturated fat component in the main algorithm

Points	Saturated fat (g/100 g)	
	Current algorithm	Proposed revision
**0**	≤1	≤1
**1**	>1	>1
**2**	>2	>2
**3**	>3	>3
**4**	>4	>4
**5**	>5	>5
**6**	>6	>6
**7**	>7	>7
**8**	>8	>8
**9**	>9	>9
**10**	>10	>10
**11**		>12
**12**		>14
**13**		>16
**14**		>18
**15**		>20

**Fig. 3:**
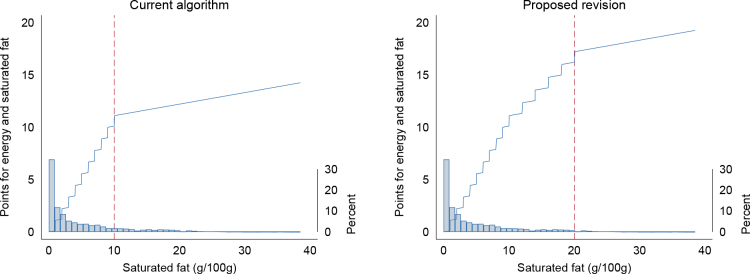
Association between saturated fat content (g/100 g) and Nutri-Score points for saturated fat and energy in the Nutri-Score 2023 main algorithm (left) and the effect of the proposed modification of the scale for saturated fat (right). The histograms indicate the distribution of saturated fat in products in the Tradesolution and Until databases, categorised in the main algorithm (*N* = 25,833).

When applying the suggested revision, a high content of saturated fat was penalised with more unfavourable points (Fig. 3).

**Fig. 4:**
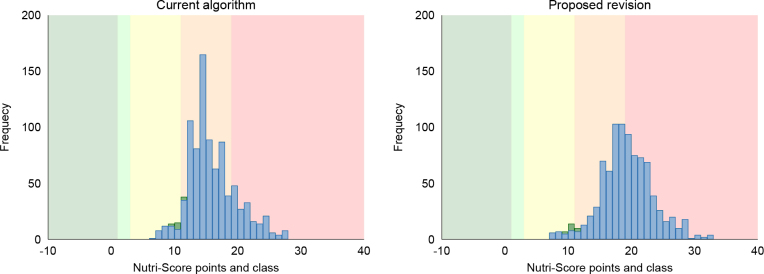
Total Nutri-Score points and class in cheese with saturated fat content ≥10 g/100 g before (left) and after (right) modifying the scale for saturated fat in the main algorithm. Frequency is the number of products. The databases are Tradesolution and Unil (*N* = 936). Green bars represents products fulfilling the criteria for the Keyhole label, and blue bars represent products not fulfilling the Keyhole criteria (21).

Of the products in the main algorithm, 3,319 products had ≥10 g saturated fat/100 g and were scored using the revised scale for saturated fat (Table 8, Fig. 4). The products were sweet snacks and desserts (*N* = 1,433, 5%), cheese (*N* = 936, 3%) and meat products (*N* = 806, 3%) with a high-saturated-fat content (range 10–64 g/100 g). However, only 1.4% (*N* = 371) of all foods obtained a new and more unfavourable Nutri-Score, mainly cheeses and cheese analogues with extra high-fat content (>30%). No Keyhole labelled cheeses were affected by the revision.

**Table 8:** Distribution (%) of Nutri-Score of the target food groups with ≥10 g saturated fat/100 g (*N* = 3,319) in the current (left) and proposed revision (right) in the main algorithm

Food group	N	Nutri-Score (%) Current algorithm						Nutri-Score (%) Proposed revision					
		NS	A	B	C	D	E	NS	A	B	C	D	E
Cheese <18% fat	39	3.7	0	0	46	41	13	3.7	0	0	46	41	13
Cheese 18–29% fat	478	4.2	0	0	7	69	24	4.3	0	0	3	61	36
Cheese >29% fat	419	4.1	0	0	0	86	14	4.7	0	0	0	29	71
Plant-based cheese analogue	28	4.1	7	7	4	25	56	4.3	7	7	4	13	69
Sweet snacks and desserts	1,433	4.5	2	1	9	23	65	4.5	2	1	9	22	66
Meat products	806	3.7	14	3	17	34	32	3.7	14	3	17	34	32
Biscuits and cakes	68	4.0	1	1	24	45	29	4.0	1	1	24	44	30
Ready meals	13	3.1	4	12	60	20	4	3.1	4	12	60	20	4
Salty snacks	23	4.1	1	1	10	64	24	4.1	1	1	10	64	24

NS, Mean Nutri-Score (A-E = 1–5). Lower value indicates higher nutritional quality. The databases are Tradesolution and Unil.

#### Including all products with high-fat content in the algorithm for high-fat foods

**Fig. 5:**
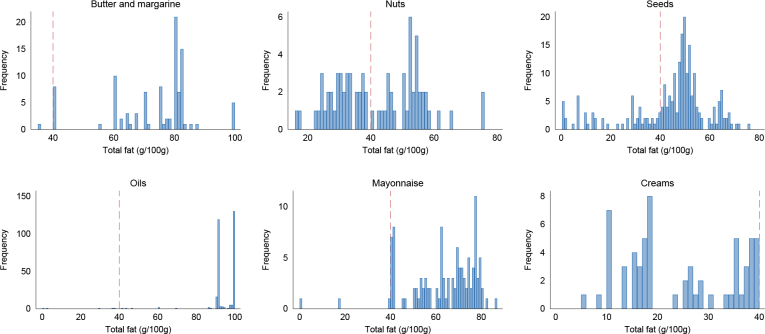
Total fat content in butter and margarine (*N* = 101), nuts (*N* = 82), seeds (*N* = 232), oils (*N* = 307), mayonnaise (*N* = 117) and creams (*N* = 63). Dotted red line indicates 40 g fat/100 g. Frequency is the number of products. The databases are Tradesolution and Unil.

Henceforth, we refer to the proposed revised algorithm for fats, oils, nuts and seeds as *the algorithm for high-fat foods*. As for carbohydrate-rich foods, we first identified a cut-off to define high-fat products. When inspecting the total fat content, most products in the fats, oils, nuts and seeds algorithm had a total fat content of ≥40 g/100 g (Fig. 5). Most mayonnaise also had a fat content above this cut-off, whilst creams were below. Thus, this was selected as the cut-off to define high-fat foods. In the databases, 1,631 (6%) products had a total fat content of ≥40 g/100 g. Therefore, for products with ≥40 g fat/100 g, the algorithm for high-fat foods should be applied.

In the databases, 777 products (3%) qualified for the Nutri-Score 2023 algorithm for fats, oils, nuts and seeds. Of the 25,833 products that are included in the main algorithm for general foods, 987 products (4%) were moved to the algorithm for high-fat foods in this suggested revision (Table 9), mainly mayonnaise products (*N* = 114), dressings (*N* = 314), some cheeses (*N* = 67) and sweet snacks and desserts (*N* = 194). When the cut-off at ≥40 g fat/100 g was applied, 144 products were moved from the algorithm for high-fat foods to the main algorithm for general foods (Table 10).

**Table 9:** Distribution (%) of the target food groups with ≥40 g total fat (*N* = 987) in the main algorithm (left) and after moving to the algorithm for high-fat foods (right)

Food group	N	Nutri-Score (%) Current algorithm						Nutri-Score (%) Proposed revision					
		NS	A	B	C	D	E	NS	A	B	C	D	E
Mayonnaise	114	4.1	0	0	14	66	20	2.9	0	14	83	3	0
Sauce and dressing	314	4.2	0	0	6	53	41	3.2	0	8	57	21	14
Cheese	67	4.4	0	0	0	58	42	4.5	0	0	18	15	67
Creams**^a^**	2	5.0	0	0	0	0	100	5.0	0	0	0	0	100
Meat products	228	5.0	0	0	0	4	96	4.8	0	0	2	19	79
Sweet snacks and desserts	209	4.8	0	0	1	14	85	4.7	0	1	6	14	79

NS, Mean Nutri-Score (A-E = 1–5). Lower value indicates higher nutritional quality. The databases are Tradesolution and Unil.

a Previously in the algorithm for fats, oils, nuts and seeds.

**Table 10:** Distribution (%) of Nutri-Score of the target food groups (*N* = 144) with <40 g fat/100 g, in the algorithm for fats (left) and after moving to the main algorithm (right)

Food group	N	Nutri-Score (%) Current algorithm						Nutri-Score (%) Proposed revision					
		NS	A	B	C	D	E	NS	A	B	C	D	E
Creams	42	3.8	0	3	3	92	1	3.7	0	3	20	77	0
Plant-based cream analogue	21	3.5	0	32	4	50	14	3.7	0	5	38	38	19
Coconut cream	7	4.0	0	0	0	100	0	4.0	0	0	0	100	0
Nuts and seeds	68	3.0	16	9	38	34	3	3.8	19	0	3	41	37
Margarine	1	3.0	0	0	100	0	0	4.0	0	0	0	100	0
Sauce and dressing	5	4.0	0	20	20	0	60	4.3	20	0	20	0	60

NS, Mean Nutri-Score (A-E = 1–5). Lower value indicates higher nutritional quality. The databases are Tradesolution and Unil.

**Fig. 6:**
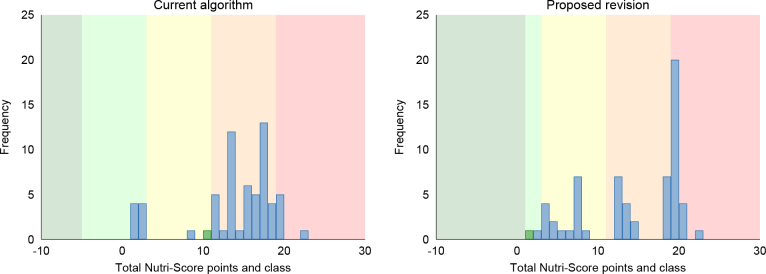
Total Nutri-Score points and class in creams in the algorithm for fats (left) and after moving creams to the algorithm for general foods and when the new scale for saturated fat is applied (right). Frequency is the number of products. The databases are Tradesolution and Unil (*N* = 63). Green bars represent products fulfilling the criteria for the Keyhole label, and blue bars represent products not fulfilling the Keyhole criteria (21).

Most creams (96%) do not qualify for the algorithm for high-fat foods. Thus, creams with <40 g fat/100 g are moved to the main algorithm for general foods. When the new proposed scale for the saturated fat was applied in the main algorithm for general foods, the variation in nutritional score for creams was greatly increased (Fig. 6). The proposed revision allowed for separation between low, moderate and high-fat cream products. After the revision, low-fat cream products with ≤10% fat got a Nutri-Score B or C, and products with 18–37% fat get Nutri-Score D, whilst products with the highest fat content (>37%) got Nutri-Score E. Creams with 40 g fat/100 g that remained in the algorithm for high-fat foods also got an E.

#### Rewarding oils high in unsaturated fat in the algorithm for high-fat foods

When inspecting the content of saturated fat in plant-based oils, most tropical oils had a saturated fatty acids content ≥25 g/100 g, whilst other plant-based oils had a saturated fatty acids content lower than this. We suggest giving favourable points for a low content of saturated fat in products with ≥90% fat (Table 11), which, in practice, are oils. This will replace the existing FVL-rule for oils.

**Table 11:** The suggested new favourable component for products with ≥90% fat in the algorithm for high-fat foods, replacing the FVL-component

Points	Saturated fatty acids (g/100 g)
0	≥25
3	<25

In total, 56 of the 302 oils in the dataset were affected by the revision, and most plant-based oils get Nutri-Score B, whilst tropical oils get Nutri-Score D or E (Table 12).

**Table 12:** Distribution (%) of Nutri-Score of the target food groups with ≥90% fat (*N* = 302) in the current (left) and proposed revision (right) in the algorithm for high-fat foods

Food group	N	Nutri-Score (%) Current algorithm						Nutri-Score (%) Proposed revision					
		NS	A	B	C	D	E	NS	A	B	C	D	E
Olive oil	169	2.0	0	99	1	0	0	2.1	0	90	9	1	0
Rapeseed oil	41	2.2	0	85	12	0	3	2.1	0	93	5	0	2
Coconut and palm oil	14	4.6	0	7	0	14	79	4.6	0	7	0	14	79
Other plant-based oils	78	2.8	0	26	69	3	2	2.3	0	79	15	3	2

NS, Mean Nutri-Score (A-E = 1–5). Lower value indicates higher nutritional quality. The databases are Tradesolution and Unil.

### Implementation

All proposed revisions were targeted towards specific food groups and only affected those intended to. If all proposed revisions are implemented in the databases, 2,846 (11%) products are moved to a different class, of which 3% are moved to a better class and 8% to a poorer class. The proposed revisions are briefly summarised in Table 13 and presented as the complete algorithms in Additional file 3.

**Table 13:** Proposed revisions to the Nutri-Score algorithms

Identified challenges	Proposed revision	Example of affected products	% of affected products in the Tradesolution and Unil databases *N* = 26,610
Whole grain	Penalise a low-fibre content in high-carbohydrate foods.	Refined grain products, biscuits, jams, snacks.	2.5%
Sugar	Modify the intervals and range of the sugar scale.	Desserts, cakes, jams, snacks, cereals.	3%
Fruit and vegetables	Assign all unprocessed fruits and vegetables with Nutri-Score A.	All unprocessed fruits and vegetables.	-*
Fish	Total Nutri-Score points for fish with a fish content ≥50% are calculated as Total unfavourable points – Total favourable points, regardless of the number of unfavourable points.	Prepared fish products, for example, smoked, canned or convenience fish products.	1%
Saturated fat	Modify the range of the saturated fat scale.	High-fat cheese (including plant-based).	1.4%
High-fat foods	All foods with a total fat content ≥40% are scored with the algorithm for high-fat foods, whilst all foods with a total fat content <40% are scored with the main algorithm for general foods.	Mayonnaise and dressings.	3%
Fat quality	The FVL component for oils is replaced with a component rewarding a saturated fat content <25%.	Oils with a high content of unsaturated fat.	0.2%

* The databases do not contain any unpackaged food items.

## Discussion

We propose a set of revisions to the Nutri-Score 2023 algorithms to improve the scoring of nutritional quality and better align with the NNR2023 and the Keyhole label. The revisions improved the ability of the Nutri-Score algorithms to discriminate between products related to carbohydrate quality, fat content and quality, and to reward the nutrient density of fish products. In addition, the algorithms were overall simplified and now also cover unprocessed fruits and vegetables. The suggested revisions were targeted and mostly affected a limited number of specific food products, and the revisions can be implemented independent of each other.

To the best of our knowledge, these are the first proposed revisions to the Nutri-Score 2023 algorithms. No other independent research group has previously systematically suggested multiple revisions to improve the overall performance of the Nutri-Score. Nutri-Score is regularly revised by the Scientific Committee of the Nutri-Score as part of its regulatory framework, and necessary modifications are implemented (15). One recent study proposed revisions to a previous version of Nutri-Score from 2017 to include whole grains as a favourable component (29). This study used a definition of whole grains from the Whole Grain Initiative (30) and showed the feasibility of including whole grain as an additional component in the score (29). However, the challenge with wholegrains is not that wholegrains are not rewarded, but rather that refined grains are not penalised.

### Proposed revisions to the Nutri-Score regarding carbohydrate quality

Our suggested cut-off of ≥40 g total carbohydrates/100 g to identify products to penalise for a low-fibre content may have limitations due to variations in water content. For instance, foods with nutrition declaration based on cooked weight, like pre-cooked pasta and rice, may have a lower carbohydrate content than the proposed cut-off and may therefore obtain a more favourable score on the original fibre scale. However, ready-to-eat products most often have added salt that contributes to a lower score, and more fibre is thus needed to achieve a Nutri-Score A. Most rice, pasta and bread typically have a carbohydrate content ≥40 g, so this limitation may have small impact.

The new fibre scale for high-carbohydrate foods aimed at increasing the discrimination between whole grain and refined grain products and allocate refined grain products to a more neutral score. This goal largely succeeded and moved most refined grain rice, pasta and flour products from Nutri-Score A to B or C. However, a minor part of the whole grain products (mostly rice and pasta) was also moved from A to B or C, due to low fibre content. Some of these products were made from a mix of whole grain and refined grain. Importantly, the databases only included five whole grain rice products, almost all with a very low fibre content. Most of these products were moved to a Nutri-Score C. However, there are whole grain rice products with a fibre content >4.1 g/100 g, which were not accounted for in our databases, which have the potential to receive Nutri-Score A. Penalising a poor carbohydrate quality was inspired by its successful implementation in other nutrient profiling systems, such as the Food Compass (31) and other carbohydrate quality metrics (32).

In the Nutri-Score 2023 algorithms, foods receive favourable points for having a relatively low fibre content, starting from 3 g/100 g. This aligns with the regulation for ‘source of fiber’ claim in the EU (33). Our proposed revision would penalise some foods, which may bear this claim. However, we view this not as a contradiction but rather as a complementary information. The fibre content claim and the Nutri-Score offer distinct information to consumers and can coexist in the market.

Using fibre as a favourable component in the Nutri-Score could potentially increase the risk of food producers, adding low-quality fibre to their products solely to improve their score. An alternative solution would be to only reward naturally occurring fibre or including whole grain as a component. This would, however, require definitions of what is considered naturally occurring fibre or whole grain. As of now, uniform definitions of these components are not in place in the EU. Including whole grain as a component in the score would reduce the risk of poor-quality product development. Furthermore, increased whole grain consumption is included in the dietary guidelines in most European countries (34). We acknowledge the significance of including whole grain as a component in Nutri-Score. Once a consistent definition of whole grain is established, we advocate for a review of the Nutri-Score to incorporate whole grain. The use of fibre as a component requires monitoring to ensure high-quality product development.

The revision of the sugar scale aimed at moving sugar-rich products to a poorer score and was successful. Although breakfast cereals were the target foods, a lot of other foods with a high-total sugar content (>12 g/100 g) were also affected by the revision and got a poorer Nutri-Score. The revision to a more compressed sugar scale for total sugar is in line with the nutritional recommendation in most European countries (34) as well as the European Food Safety Authority (EFSA), stating that the intake of sugars should be limited (35). The primary sources of added sugar in European countries are mainly found within the food groups of ‘sugar and confectionery’ and also fine bakery products (35, 36), which were all targeted by the revised sugar scale. The proposed sugar scale has an interval of 2 g total sugar per point above 12 g total sugar/100 g. Equally, the sugar scale in the current algorithm for beverages also has different increments throughout the scale, adjusted not to penalise the sugar naturally present in milk products (19). Whilst some dried fruits and products containing dried fruits were negatively affected by the revised sugar scale, dried fruits without added sugar were mainly unaffected by the revision. As we also introduced a Nutri-Score for unprocessed fruits and vegetables, the revised sugar scale had no impact on fresh fruits and vegetables.

In the Nutri-Score 2023 algorithms, total sugar is included as an unfavourable component since other forms of sugars are not specified on the nutrient declaration. Added/free sugars have been associated with conditions such as obesity, dyslipidaemia and type 2 diabetes, whilst the connection between total sugar and diet-related non-communicable diseases is less clear (36). Therefore, using added/free sugar as a component in Nutri-Score instead of total sugar would be more appropriate, but unfortunately, this information is currently unavailable on nutritional labels, and a clear definition is lacking. However, the FVL and protein components partly compensate the unfavourable points which some healthy foods, such as fruits and dairy, receive for total sugar. Therefore, swapping total sugar for added/free sugar may not have a significant impact in practice.

### Proposed revisions to the Nutri-Score regarding fish

The proposed revision of fish aimed at encouraging increased consumption of fish and removing the protein cap for fish succeeded in moving almost all Keyhole eligible fish products from E or D to a better score. The current Nutri-Score algorithm has the same rule (removal of the protein cap) for cheeses, to ensure that the protein content (as a proxy for calcium content) in cheese is rewarded. Similarly, the removal of the protein cap for fish ensures that the amount of omega-3 fatty acids, iodine, selenium and vitamin D in fish is accounted for. Compared with the Nutri-Score, the Food Compass includes more indicators and rewards the nutritional quality of fish even more (31), as it imposes a comparatively lower penalty on the salt content present in fish. Similarly, the Keyhole label also has a high allowance for salt in fish products. The intake of fish in Norway (37, 38) and other Nordic countries (39, 40) is lower than recommended by the NNR2023 (recommended intake 300-450g/week) (20). The proposed revision may promote an increased fish consumption, which is encouraged in the dietary guidelines of most countries within the EU (41). Whilst fish may contain environmental toxins, the benefits of increased consumption outweigh the potential negative health risks (42).

### Proposed revisions to the Nutri-Score regarding fat quality

The revised saturated fat component along with keeping all foods with <40 g/100 g fat in the main algorithm aimed towards better differentiation between products with high amount of saturated fat, and mainly affected high-fat cheese and dairy (such as creams). Both cheese and creams previously clustered in Nutri-Score D, but with the proposed revisions, the discrimination between products with different saturated fat content was improved, and products with the highest saturated fat content received a poorer score. This is an important aspect from a public health standpoint and consistent with the dietary guidelines of most countries within EU (43), as well as the NNR2023, which highlight that substituting saturated fat with unsaturated fat improves blood lipids, reduces the risk of cardiovascular disease and improves glucose regulation (20). The nutrient profiling system The Health Star Rating penalises up to 90 g saturated fat/100 g in the corresponding main foods algorithm (28).

The cut-off at ≥40 g fat/100 g as cut-off for inclusion in the algorithm for high-fat foods is an important simplification of the algorithms. Currently, inclusion in the algorithm for fats, oils, nuts and seeds is based on defined food categories. This leads to some foods with a low fat content (such as low-fat cream products) getting a poor score regardless of the low fat content, and some high-fat foods (such as mayonnaise) getting a poor score in the main algorithm regardless of fat quality. This food category-based selection into the different algorithms does not provide the best classification of nutritional quality of foods. Our proposed revision allows for products with similar fat and high energy contents to be classified with the same algorithm and, therefore, allows for a better classification of nutritional quality. Using ≥40 g fat/100 g as the cut-off moved the creams to the main algorithm for general foods, and apart from that, only one other product (a low-fat margarine) also moved from the algorithm for high-fat foods and received a poorer score than it would have got in the algorithm for high-fat foods (moved from C to D). We also propose another important simplification of the algorithm for high-fat foods through replacement of the favourable FVL-component for oils with a new fat-quality component for products with ≥90% fat. Similarly, this revision provides a better classification of nutritional quality and more in line with the NNR2023.

### Implications and generalisability

Our proposed revisions to the Nutri-Score 2023 algorithms, although modest in scale, represent refinements achieved through adjusted components and simplifications. The adjusted components were the scales for fibre (including a cut-off for high-carbohydrate foods), sugar and saturated fat. In the Nutri-Score 2023 algorithms, these scales were based on reference values from the EU food information to consumers regulation (5). However, as previous papers have shown, the scales were insufficient in capturing variation in these components within some food groups. Therefore, we propose new cut-offs and thresholds based on the variation amongst the majority of food products in the market. The proposed revised scales increased the variation in scores within targeted food categories, which potentially leads to better guidance towards healthier food choices. Furthermore, in the Nutri-Score 2023 algorithms, some foods with a high fat content, such as mayonnaise and dressings, are scored with the main algorithm for general foods, whilst other foods with a lower fat content, such as cream (including low-fat alternatives), are scored with the algorithm for fats, oils, nuts and seeds. This algorithm classification by food categories is neither intuitive nor logical. In addition, the FVL-component for oils in the Nutri-Score algorithm is another exception rule, which can cause confusion amongst producers, and it does not reflect nutritional recommendations. The proposed ≥40 g fat/100 g as cut-off for inclusion in the algorithm for high-fat foods and the new fat quality component in high-fat products thus represent important simplifications of the algorithms.

Taken together, the proposed revisions adhere to the predefined framework of the NewTools-project and represent a significant step towards enhancing the relevance of the Nutri-Score for a broader range of countries. Recommendations to reduce the intake of saturated fat and sugar to replace saturated fat with unsaturated fat and refined grains with whole grains are consistent with the dietary guidelines of most European countries (34). The poor discriminatory ability of the Nutri-Score 2023 for whole grain and refined grain products has also been acknowledged as a weakness by the scientific committee of the Nutri-Score (10, 15). Consequently, all the proposed revisions in this paper hold relevance for other European countries. However, it is important to note that our evaluation of the Nutri-Score has been limited to a Norwegian context, and we have not assessed the impact of the modifications in other food markets.

### Future studies

Previous research has found associations between a dietary quality index derived from the Nutri-Score nutrient profiling system and mortality (44), emphasising the relevance of using Nutri-Score as an important public health policy instrument. However, it is crucial to verify the continued association between the Nutri-Score system and its impact on health as the system develops. The association between the Nutri-Score 2023 algorithms and our proposed revised Nutri-Score, and diet quality indices and health outcomes will further be tested in the NewTools project.

Within the NewTools framework of the nutritional score, it is specified that the score is not intended to encompass all aspects of nutritional quality but rather serve as a complementary tool to dietary guidelines. The scientific committee of the Nutri-Score has also acknowledged this intention with Nutri-Score (15). However, continuous evaluation and development is crucial to ensure that the score reflects important nutritional aspects.

A limitation of the Nutri-Score becomes apparent when dealing with mixed dishes and ready meals, as the score fails to capture meaningful variations in nutritional content. When scoring systems evaluate nutrient contents per 100 g, the scoring is significantly influenced by the food’s water content (11, 31, 45). Ready meals often have low energy content due to their high content of water. Despite the potential for vastly different nutritional quality of ready meals, this variability remains largely unaccounted for due to the system’s reliance on scoring per 100 g, and most ready meals in Norway obtain Nutri-Score C. This represents a remaining challenge with the Nutri-Score, which may be considered in future revisions of the algorithms.

### Strengths and limitations

A strength of the current study was the broad Nordic perspective of the research group and the rigorous work on which the current study is based, using diverse methods to capture all the potential challenges to the Nutri-Score 2023 algorithms in a Norwegian setting (23, 24). An additional strength was the availability of two food databases comprising most pre-packed foods on the Norwegian market.

One limitation of the study is that the evaluation of our proposed revisions of the Nutri-Score was limited to a Norwegian context, and the impact in other food markets is unknown. Furthermore, we identified some implausible data in the databases, but there might be residual unidentified errors. Thus, the number of foods affected by the proposed revisions might be slightly greater or smaller than estimated. The lack of data on fibre in the database (62%) was another limitation, but all foods with a high carbohydrate content had data on fibre.

## Conclusion

Our proposed revisions to the Nutri-Score 2023 algorithms enhanced the alignment with the nutritional evidence of the NNR2023 and the Keyhole label. These revisions improved the discrimination related to carbohydrate quality, fat content and quality, and enhanced the scoring of certain fish products. Furthermore, we suggest some simplifications of the algorithm and propose the inclusion of unprocessed fruits and vegetables. These targeted revisions may contribute to the refinement and effectiveness of the Nutri-Score algorithm in guiding consumers towards healthier food choices. With this study, we hope that the proposed revisions can be further tested for alignment in other countries, which have adopted the Nutri-Score label, and be considered in the next revision of the Nutri-Score.

## Supplementary Material



## Declarations

### Availability of data and materials

The data that support the findings of this study are available from Tradesolution and NorgesGruppen (Unil), but restrictions apply to the availability of these data, which were used under licence for the current study, and so are not publicly available. Data may however be available from the authors upon reasonable request and with permission of Tradesolution and NorgesGruppen. Data from Tradesolution are publicly available from vetduat.no.
