# Supplementary material for: Improvements to the Nutri-Score to address challenges identified in a Nordic setting
Source: Food Nutr Res. 2025 Jan 29;69:10.29219/fnr.v69.10914. doi: 10.29219/fnr.v69.10914 (PMC11836775; doi:10.29219/fnr.v69.10914)
Supplement: Supplementary file 1 [file FNR-69-10914-s1.docx]

**Supplementary material**

***Additional file 1. The Nutri-Score 2023 algorithms.***

To calculate the total score for the main algorithm for general foods, summarize the points from the unfavorable components (Table 1).

| **Table 1. Nutri-Score main algorithm for general foods** | | | | | | | |
| --- | --- | --- | --- | --- | --- | --- | --- |
| Points | Unfavorable components | | | | Favorable components | | |
|  | Energy  (kJ/100g) | Sugars (g/100g) | Saturated fat (g/100g) | Salt  (g/100g) | Protein*  (g/100g) | Fiber  (g/100g) | Fruit, vegetables, legumes (%) |
| 0 | ≤ 335 | ≤ 3.4 | ≤ 1.0 | ≤ 0.2 | ≤ 2.4 | ≤ 3.0 | ≤ 40 |
| 1 | > 335 | > 3.4 | > 1.0 | > 0.2 | > 2.4 | > 3.0 | > 40 |
| 2 | > 670 | > 6.8 | > 2.0 | > 0.4 | > 4.8 | > 4.1 | > 60 |
| 3 | > 1005 | > 10 | > 3.0 | > 0.6 | > 7.2 | > 5.2 | - |
| 4 | > 1340 | > 14 | > 4.0 | > 0.8 | > 9.6 | > 6.3 | - |
| 5 | > 1675 | > 17 | > 5.0 | > 1.0 | > 12 | > 7.4 | > 80 |
| 6 | > 2010 | > 20 | > 6.0 | > 1.2 | > 14 |  |  |
| 7 | > 2345 | > 24 | > 7.0 | > 1.4 | > 17 |  |  |
| 8 | > 2680 | > 27 | > 8.0 | > 1.6 |  |  |  |
| 9 | > 3015 | > 31 | > 9.0 | > 1.8 |  |  |  |
| 10 | > 3350 | > 34 | > 10 | > 2.0 |  |  |  |
| 11 |  | > 37 |  | > 2.2 |  |  |  |
| 12 |  | > 41 |  | > 2.4 |  |  |  |
| 13 |  | > 44 |  | > 2.6 |  |  |  |
| 14 |  | > 48 |  | > 2.8 |  |  |  |
| 15 |  | > 51 |  | > 3.0 |  |  |  |
| 16 |  |  |  | > 3.2 |  |  |  |
| 17 |  |  |  | > 3.4 |  |  |  |
| 18 |  |  |  | > 3.6 |  |  |  |
| 19 |  |  |  | > 3.8 |  |  |  |
| 20 |  |  |  | > 4.0 |  |  |  |
| *Red meat products are given maximum 2 protein points. | | | | | | | |

If unfavorable component is ≥ 11 points, then apply formula: Nutri-Score points = total unfavorable components points - (points from the fiber component + points from the fruit, vegetables, and legumes component). If unfavorable component is < 11 points or the food is cheese, then apply formula: Nutri-Score points = total unfavorable component points - total favorable components points. Classify Nutri-Score using Table 2.

| **Table 2.** **Thresholds for the three Nutri-Score algorithms.** | | | | |
| --- | --- | --- | --- | --- |
| **Nutri-Score** | **Color** | **Algorithm for general foods** | **Algorithm for fats** | **Algorithm for beverages** |
| A | Dark green | Min to 0 | Min to -6 | Water |
| B | Light green | 1 to 2 | -5 to 2 | Min to 2 |
| C | Yellow | 3 to 10 | 3 to 10 | 3 to 6 |
| D | Light orange | 11 to 18 | 11 to 18 | 7 to 9 |
| E | Dark orange | 19 to max | 19 to max | 10 to max |

To calculate the total score for the algorithm for fats, oils, nuts, and seeds, summarize points from unfavorable components (Table 3).

| **Table 3. Nutri-Score total points for fats, oils, nuts, and seeds** | | | | | | | |
| --- | --- | --- | --- | --- | --- | --- | --- |
| Points | Unfavorable components | | | | Favorable components | | |
|  | Energy from saturated fat  (kJ/100g)* | Sugars  (g/100g) | Saturated fat/total fat (%) | Salt  (g/100g) | Protein (g/100g) | Fiber (g/100g) | Fruit, vegetables, legumes (%) |
| 0 | ≤ 120 | ≤ 3.4 | < 10 | ≤ 0.2 | ≤ 2.4 | ≤ 3.0 | ≤ 40 |
| 1 | > 120 | > 3.4 | < 16 | > 0.2 | > 2.4 | > 3.0 | > 40 |
| 2 | > 240 | > 6.8 | < 22 | > 0.4 | > 4.8 | > 4.1 | > 60 |
| 3 | > 360 | > 10 | < 28 | > 0.6 | > 7.2 | > 5.2 | - |
| 4 | > 480 | > 14 | < 34 | > 0.8 | > 9.6 | > 6.3 | - |
| 5 | > 600 | > 17 | < 40 | > 1.0 | > 12 | > 7.4 | > 80 |
| 6 | > 720 | > 20 | < 46 | > 1.2 | > 14 |  |  |
| 7 | > 840 | > 24 | < 52 | > 1.4 | > 17 |  |  |
| 8 | > 960 | > 27 | < 58 | > 1.6 |  |  |  |
| 9 | > 1080 | > 31 | < 64 | > 1.8 |  |  |  |
| 10 | > 1200 | > 34 | ≥ 64 | > 2.0 |  |  |  |
| 11 |  | > 37 |  | > 2.2 |  |  |  |
| 12 |  | > 41 |  | > 2.4 |  |  |  |
| 13 |  | > 44 |  | > 2.6 |  |  |  |
| 14 |  | > 48 |  | > 2.8 |  |  |  |
| 15 |  | > 51 |  | > 3.0 |  |  |  |
| 16 |  |  |  | > 3.2 |  |  |  |
| 17 |  |  |  | > 3.4 |  |  |  |
| 18 |  |  |  | > 3.6 |  |  |  |
| 19 |  |  |  | > 3.8 |  |  |  |
| 20 |  |  |  | > 4.0 |  |  |  |
| *Energy from saturated fat = saturated fat (g per 100 grams) x 37 | | | | | | | |

If unfavorable component is ≥ 7 points, then apply formula: Nutri-Score points = total unfavorable components points - (points from the fiber component + points from the fruit, vegetables, and legumes component). If unfavorable component is < 7 points, then apply formula: Nutri-Score points = total unfavorable component points – total favorable components points. Classify Nutri-Score using Table 2.

To calculate the total score for the algorithm for beverages, summarize points from unfavorable components and favorable components separately (Table 4). Nutri-Score points = total unfavorable component points – total favorable components points. Classify Nutri-Score using Table 2.

| **Table 4. Nutri-Score total points for beverages** | | | | | | | | |
| --- | --- | --- | --- | --- | --- | --- | --- | --- |
| Points | Unfavorable components | | | | | Favorable components | | |
|  | Energy (kJ/100ml) | Sugars (g/100ml) | Saturated fat (g/100ml) | Salt  (g/100ml) | NNS | Protein (g/100ml) | Fiber  (g/100ml) | Fruit, vegetables, legumes (%) |
| 0 | ≤ 30 | ≤ 0.5 | ≤ 1.0 | ≤ 0.2 |  | ≤ 1.2 | ≤ 3.0 | ≤ 40 |
| 1 | ≤ 90 | ≤ 2.0 | > 1.0 | > 0.2 |  | > 1.2 | > 3.0 | - |
| 2 | ≤ 150 | ≤ 3.5 | > 2.0 | > 0.4 |  | > 1.5 | > 4.1 | > 40 |
| 3 | ≤ 210 | ≤ 5.0 | > 3.0 | > 0.6 |  | > 1.8 | > 5.2 | - |
| 4 | ≤ 240 | ≤ 6.0 | > 4.0 | > 0.8 | Presence | > 2.1 | > 6.3 | > 60 |
| 5 | ≤ 270 | ≤ 7.0 | > 5.0 | > 1.0 |  | > 2.4 | > 7.4 | - |
| 6 | ≤ 300 | ≤ 8.0 | > 6.0 | > 1.2 |  | > 2.7 |  | > 80 |
| 7 | ≤ 330 | ≤ 9.0 | > 7.0 | > 1.4 |  | > 3.0 |  |  |
| 8 | ≤ 360 | ≤ 10 | > 8.0 | > 1.6 |  |  |  |  |
| 9 | ≤ 390 | ≤ 11 | > 9.0 | > 1.8 |  |  |  |  |
| 10 | > 390 | > 11 | > 10 | > 2.0 |  |  |  |  |
| 11 |  |  |  | > 2.2 |  |  |  |  |
| 12 |  |  |  | > 2.4 |  |  |  |  |
| 13 |  |  |  | > 2.6 |  |  |  |  |
| 14 |  |  |  | > 2.8 |  |  |  |  |
| 15 |  |  |  | > 3.0 |  |  |  |  |
| 16 |  |  |  | > 3.2 |  |  |  |  |
| 17 |  |  |  | > 3.4 |  |  |  |  |
| 18 |  |  |  | > 3.6 |  |  |  |  |
| 19 |  |  |  | > 3.8 |  |  |  |  |
| 20 |  |  |  | > 4.0 |  |  |  |  |

NNS, Non-nutritive sweeteners

***Additional file 2.docx*
Table 1. Distribution (%) of the food groups not targeted (<40g carbohydrates/100g, n=2484) in the main algorithm for general foods.**

| Food group |  | Nutri-Score (%) Current algorithm | | | | | |
| --- | --- | --- | --- | --- | --- | --- | --- |
|  | **N** | **NS** | **A** | **B** | **C** | **D** | **E** |
| Whole grain bread ^a^ | 14 | 1.4 | 70 | 20 | 10 | 0 | 0 |
| Refined grain bread | 35 | 2.6 | 20 | 23 | 28 | 29 | 0 |
| Other breads | 11 | 2.2 | 9 | 73 | 9 | 9 | 0 |
| Whole grain rice ^a^ | 2 | 1.5 | 50 | 50 | 0 | 0 | 0 |
| White rice | 29 | 1.7 | 42 | 48 | 10 | 0 | 0 |
| Whole grain pasta ^a^ | 1 | 1 | 100 | 0 | 0 | 0 | 0 |
| White pasta | 54 | 3.0 | 2 | 9 | 78 | 11 | 0 |
| Whole grain flour ^a^ | 0 | - | - | - | - | - | - |
| Refined flour | 3 | 2.5 | 33 | 0 | 67 | 0 | 0 |
| Breakfast cereals | 34 | 3.1 | 6 | 21 | 41 | 23 | 9 |
| Quinoa, bulgur, couscous | 0 | - | - | - | - | - | - |
| Sweet snacks and desserts | 423 | 3.8 | 7 | 1 | 22 | 41 | 29 |
| Biscuits, cakes, pastries, muslibars | 87 | 3.8 | 2 | 0 | 26 | 52 | 20 |
| Sandwich toppings (jam etc.) | 104 | 2.9 | 19 | 4 | 45 | 30 | 2 |
| Fruit and berries ^b^ | 94 | 1.4 | 76 | 7 | 16 | 1 | 0 |
| Legumes ^c^ | 600 | 1.7 | 71 | 6 | 11 | 10 | 2 |
| Sauce and dressing | 458 | 3.7 | 3 | 4 | 40 | 29 | 24 |
| Salty snacks | 6 | 4.7 | 0 | 0 | 0 | 33 | 67 |
| Ready meals | 529 | 3.0 | 3 | 13 | 64 | 19 | 1 |

NS, Mean Nutri-Score points (A-E=1-5).
a Whole grain defined as the first ingredient being a whole grain flour or being eligible for the Keyhole label.
b Contains dried and canned fruit and berries
c Contain dried and candied legumes

***Additional file 3.docx***Proposed revisions to the Nutri-Score algorithms. The compiled algorithms with proposed revisions highlighted in light grey.

**Step 1: Foods covered by the Nutri-Score**

Except for the products already being covered by the Nutri-Score, we propose that the following also should be covered by the Nutri-Score.

- Fresh and minimally processed fruit and vegetables

All whole fresh fruit (except coconut) and vegetables, fungi and legumes (except peanuts) as sold with no processing, plus these same products that have only been peeled, cut and/or surface treated and/or blanched and/or frozen (not dried), or canned without the addition of fat, sugars/sweeteners or salt receive Nutri-Score A and are allocated with a nutritional score of -5.

**Step 2: Determine the Nutri-Score algorithm of the product**

There are three Nutri-Score algorithms:

| - Beverages | All beverages intended to be drunk, such as fruit or vegetable juices, soft drinks, milk, milk-based beverages, fermented milk-based beverages, and plant-based beverages. |
| --- | --- |
| - High-fat foods | All solid foods with a total fat content ≥40 g/100 g |
| - General foods | All solid foods, soups, and stocks, with a total fat content <40 g/100 g |

**Step 3: Calculate the Nutri-Score points**

To calculate the total score for the main algorithm for general foods, summarize points from unfavourable components (Table 1).

| **Table 1. Nutri-Score points for general foods** | | | | | | | | |
| --- | --- | --- | --- | --- | --- | --- | --- | --- |
| Points | Unfavourable components | | | | Favourable components | | | |
|  | Energy  (kJ/100g) | Sugars (g/100g) | Saturated fat (g/100g) | Salt  (g/100g) | Protein ^a^  (g/100g) | Fiber ^c^  (g/100g) | Fiber ^b^  (g/100g) | FVL (%) |
| -3 | - | - | - | - | - |  | ≤ 2.0 | - |
| -2 | - | - | - | - | - |  | ≤ 2 | - |
| 0 | ≤ 335 | ≤ 3.4 | ≤ 1.0 | ≤ 0.2 | ≤ 2.4 | ≤ 3.0 | - | ≤ 40 |
| 1 | > 335 | > 3.4 | > 1.0 | > 0.2 | > 2.4 | > 3.0 | - | > 40 |
| 2 | > 670 | > 6.8 | > 2.0 | > 0.4 | > 4.8 | > 4.1 | > 4.1 | > 60 |
| 3 | > 1005 | > 10 | > 3.0 | > 0.6 | > 7.2 | > 5.2 | > 5.2 | - |
| 4 | > 1340 | > 12 | > 4.0 | > 0.8 | > 9.6 | > 6.3 | > 6.3 | - |
| 5 | > 1675 | > 14 | > 5.0 | > 1.0 | > 12 | > 7.4 | > 7.4 | > 80 |
| 6 | > 2010 | > 16 | > 6.0 | > 1.2 | > 14 |  |  |  |
| 7 | > 2345 | > 18 | > 7.0 | > 1.4 | > 17 |  |  |  |
| 8 | > 2680 | > 20 | > 8.0 | > 1.6 |  |  |  |  |
| 9 | > 3015 | > 22 | > 9.0 | > 1.8 |  |  |  |  |
| 10 | > 3350 | > 24 | > 10 | > 2.0 |  |  |  |  |
| 11 |  | > 26 | > 12 | > 2.2 |  |  |  |  |
| 12 |  | > 28 | > 14 | > 2.4 |  |  |  |  |
| 13 |  | > 30 | > 16 | > 2.6 |  |  |  |  |
| 14 |  | > 32 | > 18 | > 2.8 |  |  |  |  |
| 15 |  | > 34 | > 20 | > 3.0 |  |  |  |  |
| 16 |  |  |  | > 3.2 |  |  |  |  |
| 17 |  |  |  | > 3.4 |  |  |  |  |
| 18 |  |  |  | > 3.6 |  |  |  |  |
| 19 |  |  |  | > 3.8 |  |  |  |  |
| 20 |  |  |  | > 4.0 |  |  |  |  |
| a Red meat products are given maximum 2 protein points. b Fibre points for foods with a total carbohydrate content (including fibre) <40 g/100 g.  c Fibre points for foods with a total carbohydrate content (including fibre) ≥40 g/100 g. FVL; Fruit, vegetables, legumes | | | | | | | | |

If unfavourable component is ≥ 11 points, then apply formula: Nutri-Score points = total unfavourable components points - (points from the fibre component + points from the fruit, vegetables, and legumes component). If unfavourable component is < 11 points or the food is cheese or contain >=50% fish, then apply formula: Nutri-Score points = total unfavourable component points - total favourable components points. Classify Nutri-Score using Table 2.

| **Table 2.** **Thresholds for the Nutri-Score algorithms.** | | | | |
| --- | --- | --- | --- | --- |
| **Nutri-Score** | **Colour** | **Algorithm for general foods** | **Algorithm for high-fat foods** | **Algorithm for beverages** |
| A | Dark green | Min to 0 | Min to -6 | Water |
| B | Light green | 1 to 2 | -5 to 2 | Min to 2 |
| C | Yellow | 3 to 10 | 3 to 10 | 3 to 6 |
| D | Light orange | 11 to 18 | 11 to 18 | 7 to 9 |
| E | Dark orange | 19 to max | 19 to max | 10 to max |

To calculate the total score for the algorithm for high-fat foods, summarize points from unfavourable components (Table 3).

| **Table 3. Nutri-Score points for high-fat foods (total fat content ≥40 g/100 g)** | | | | | | | |
| --- | --- | --- | --- | --- | --- | --- | --- |
| Points | Unfavourable components | | | | Favourable components | | |
|  | Energy from saturated fat  (kJ/100g) ^a^ | Sugars (g/100g) | Saturated fat/total fat (%) | Salt  (g/100g) | Protein (g/100g) ^b^ | Fiber (g/100g) | Saturated fat (g/100g) ^c^ |
| 0 | ≤ 120 | ≤ 3.4 | < 10 | ≤ 0.2 | ≤ 2.4 | ≤ 3.0 | - |
| 1 | > 120 | > 3.4 | < 16 | > 0.2 | > 2.4 | > 3.0 | - |
| 2 | > 240 | > 6.8 | < 22 | > 0.4 | > 4.8 | > 4.1 | - |
| 3 | > 360 | > 10 | < 28 | > 0.6 | > 7.2 | > 5.2 | < 25 |
| 4 | > 480 | > 14 | < 34 | > 0.8 | > 9.6 | > 6.3 |  |
| 5 | > 600 | > 17 | < 40 | > 1.0 | > 12 | > 7.4 |  |
| 6 | > 720 | > 20 | < 46 | > 1.2 | > 14 |  |  |
| 7 | > 840 | > 24 | < 52 | > 1.4 | > 17 |  |  |
| 8 | > 960 | > 27 | < 58 | > 1.6 |  |  |  |
| 9 | > 1080 | > 31 | < 64 | > 1.8 |  |  |  |
| 10 | > 1200 | > 34 | ≥ 64 | > 2.0 |  |  |  |
| 11 |  | > 37 |  | > 2.2 |  |  |  |
| 12 |  | > 41 |  | > 2.4 |  |  |  |
| 13 |  | > 44 |  | > 2.6 |  |  |  |
| 14 |  | > 48 |  | > 2.8 |  |  |  |
| 15 |  | > 51 |  | > 3.0 |  |  |  |
| 16 |  |  |  | > 3.2 |  |  |  |
| 17 |  |  |  | > 3.4 |  |  |  |
| 18 |  |  |  | > 3.6 |  |  |  |
| 19 |  |  |  | > 3.8 |  |  |  |
| 20 |  |  |  | > 4.0 |  |  |  |

a Energy from saturated fat = saturated fat (g per 100 grams) x 37

b Red meat products are given maximum 2 protein points.
c Applies only to foods with ≥90% fat

If unfavourable component is ≥ 7 points, then apply formula: Nutri-Score points = total unfavourable components points - (points from the fibre component + points from the fruit, vegetables, and legumes component). If unfavourable component is < 7 points, then apply formula: Nutri-Score points = total unfavourable component points – total favourable components points. Classify Nutri-Score using Table 2.

To calculate the total score for beverages, summarize points from unfavorable components and favorable components separately (Table 4). Nutri-Score points = total unfavorable component points – total favorable components points. Classify Nutri-Score using Table 2.

| **Table 4. Nutri-Score points for beverages** | | | | | | | | |
| --- | --- | --- | --- | --- | --- | --- | --- | --- |
| Points | Unfavorable components | | | | | Favorable components | | |
|  | Energy (kJ/100ml) | Sugars (g/100 ml) | Saturated fat (g/100 ml) | Salt  (g/100 ml) | NNS | Protein (g/100ml) | Fiber  (g/100ml) | FVL (%) |
| 0 | ≤ 30 | ≤ 0.5 | ≤ 1.0 | ≤ 0.2 |  | ≤ 1.2 | ≤ 3.0 | ≤ 40 |
| 1 | ≤ 90 | ≤ 2.0 | > 1.0 | > 0.2 |  | > 1.2 | > 3.0 | - |
| 2 | ≤ 150 | ≤ 3.5 | > 2.0 | > 0.4 |  | > 1.5 | > 4.1 | > 40 |
| 3 | ≤ 210 | ≤ 5.0 | > 3.0 | > 0.6 |  | > 1.8 | > 5.2 | - |
| 4 | ≤ 240 | ≤ 6.0 | > 4.0 | > 0.8 | Presence | > 2.1 | > 6.3 | > 60 |
| 5 | ≤ 270 | ≤ 7.0 | > 5.0 | > 1.0 |  | > 2.4 | > 7.4 | - |
| 6 | ≤ 300 | ≤ 8.0 | > 6.0 | > 1.2 |  | > 2.7 |  | > 80 |
| 7 | ≤ 330 | ≤ 9.0 | > 7.0 | > 1.4 |  | > 3.0 |  |  |
| 8 | ≤ 360 | ≤ 10 | > 8.0 | > 1.6 |  |  |  |  |
| 9 | ≤ 390 | ≤ 11 | > 9.0 | > 1.8 |  |  |  |  |
| 10 | > 390 | > 11 | > 10 | > 2.0 |  |  |  |  |
| 11 |  |  |  | > 2.2 |  |  |  |  |
| 12 |  |  |  | > 2.4 |  |  |  |  |
| 13 |  |  |  | > 2.6 |  |  |  |  |
| 14 |  |  |  | > 2.8 |  |  |  |  |
| 15 |  |  |  | > 3.0 |  |  |  |  |
| 16 |  |  |  | > 3.2 |  |  |  |  |
| 17 |  |  |  | > 3.4 |  |  |  |  |
| 18 |  |  |  | > 3.6 |  |  |  |  |
| 19 |  |  |  | > 3.8 |  |  |  |  |
| 20 |  |  |  | > 4.0 |  |  |  |  |

FVL; fruits, vegetables, and legumes. NNS; Non-nutritive sweeteners
